# Occurrence and Control of Sporadic Proliferation in Growth Arrested Swiss 3T3 Feeder Cells

**DOI:** 10.1371/journal.pone.0122056

**Published:** 2015-03-23

**Authors:** Rishi Man Chugh, Madhusudan Chaturvedi, Lakshmana Kumar Yerneni

**Affiliations:** Cell Biology Laboratory, National Institute of Pathology (ICMR), New Delhi, India; San Gallicano Dermatologic Institute, ITALY

## Abstract

Growth arrested Swiss mouse embryonic 3T3 cells are used as feeders to support the growth of epidermal keratinocytes and several other target cells. The 3T3 cells have been extensively subcultured owing to their popularity and wide distribution in the world and, as a consequence selective inclusion of variants is a strong possibility in them. Inadvertently selected variants expressing innate resistance to mitomycin C may continue to proliferate even after treatment with such growth arresting agents. The failure of growth arrest can lead to a serious risk of proliferative feeder contamination in target cell cultures. In this study, we passaged Swiss 3T3 cells (CCL-92, ATCC) by different seeding densities and incubation periods. We tested the resultant cultures for differences in anchorage-independent growth, resumption of proliferation after mitomycin C treatment and occurrence of proliferative feeder contaminants in an epidermal keratinocyte co-culture system. The study revealed subculture dependent differential responses. The cultures of a particular subculture procedure displayed unique cell size distribution and disintegrated completely in 6 weeks following mitomycin C treatment, but their repeated subculture resulted in feeder regrowth as late as 11 weeks after the growth arrest. In contrast, mitomycin C failed to inhibit cell proliferation in cultures of the other subculture schemes and also in a clone that was established from a transformation focus of super-confluent culture. The resultant proliferative feeder cells contaminated the keratinocyte cultures. The anchorage-independent growth appeared in late passages as compared with the expression of mitomycin C resistance in earlier passages. The feeder regrowth was prevented by identifying a safe subculture protocol that discouraged the inclusion of resistant variants. We advocate routine anchorage-independent growth assay and absolute confirmation of feeder disintegration to qualify feeder batches and caution on the use of fetal bovine serum.

## Introduction

Large quantities of cultured epithelial autografts (CEA) for clinical use in the treatment of extensively burned patients are speedily grown from the adult epidermal keratinocytes over the growth arrested Swiss mouse embryonic 3T3 dermal fibroblasts [1]. These cells are superior in supporting the growth of other target cells as well [2, 3]. The original inactivation method involved γ-irradiation, although a more convenient option has been the treatment with mitomycin C (MC) [3]. The growth arrested 3T3 fibroblasts reportedly survived in CEA and elicited immunogenicity in recipient resulting in complete graft breakdown [4]. Reasonably the viable feeders can result either from the mitotically inactive yet surviving feeders or the proliferating ones. Although, there is evidence of proliferation in other growth arrested mouse embryonic feeders, but there are no specific studies to link the persistence of the viable 3T3 feeders with the failure of growth arrest [5].

The 3T3 cells have the potential to undergo spontaneous transformation depending on subculture, confluence state, and type and concentration of serum [6, 7]. Repeated and inconsistent passaging of cell cultures leads to the accumulation of specific transformed variants and display of altered characteristics [8]. Selective accumulation of such variants, particularly in late passage cultures of 3T3 is a strong possibility as they have been extensively subcultured due to their popularity and wide distribution through several channels in the world [8]. But indications of transformation such as loss of contact inhibition and presentation of phenotypic differences may not readily be apparent, when the transformed variants are less frequent. However, few variants with innate resistance to growth arrest may continue to even after exposure to MC. Such proliferative feeders then become visible and contaminate the target cell cultures. We hypothesize that the presence of such variants in 3T3 cell cultures is a potential cause for failure of growth arrest.

We, therefore, propose to investigate if the proliferative feeder contamination of target cells is dependent on the adopted subculture protocol for 3T3 cells and identify preventive strategies. The identified solutions can help in eliminating apprehensions on feeder dependant culture system [9], which is the most efficient and economical method to culture stem cells compared with feeder-free systems [10]. We observed that it was essential to validate each lot of the growth arrested 3T3 cells through confirmation of the complete disintegration of feeders before qualifying them as safe feeders.

## Materials & Methods

### 3T3 fibroblast culture

The frozen SWISS 3T3 cells (CCL-92, ATCC) supplied at 115^th^ passage (designated as zero passage) were quickly thawed and grown in 3T3-CBS medium consisting of DMEM with 1.5 grams of sodium bicarbonate per liter and 10% calf bovine serum (CBS) in a humidified 5% CO_2_ atmosphere at 37°C. The cells were serially subcultured until 6 passages to establish cryopreserved master and working banks (S1 Fig.). The frozen cells were incubated for 4 days to compensate for the initial slow growth while the subsequent cultures were passaged at uniform intervals of 3 days. The cultures were negative for Mycoplasma [11].

### Subculture schemes

The working bank cultures were subjected to specific subculture schemes which were determined after several rounds of preliminary experiments. Initially, 7^th^ passage (P7) cultures were setup in T75 flasks from working bank by seeding 3000 cells per cm^2^ and were subcultured by three schemes (S2 Fig.). Two of the schemes denoted as 3K3D and 3K4D, represented incubation of 3000 plated cells per cm^2^ for 3 and 4 days, respectively. The third scheme of 4K3D involved 3 days incubation of 4000 cells plated per cm^2^. The resultant cells were tested for anchorage-independent growth and stability of growth arrest by MC treatment. Further, the 3K3D cells that exhibited irreversible growth arrest and no anchorage-independent growth were repeatedly subcultured by 3K3D procedure to determine the number of passages that these characters persisted. Simultaneously, the cells were also passaged serially by the 4K3D scheme for comparison to match with the same subculture interval. The subcultures were continued in T25 flasks until the cells exhibited an obvious 3-dimensional growth in suspension.

### Generation of spontaneously transformed clone

A transformed clone of 3T3 cells was derived from a spontaneously induced transformation focus in a confluent culture (S2 Fig.) that was established by incubating 3000 cells per cm^2^ in 3T3-FBS medium containing 10% Fetal Bovine Serum [6]. The incidence of foci was increased by sub-culturing a large focus which was scraped with a cell scrapper. Subsequent culture was trypsinized and thousand cells were incubated in methyl cellulose for two weeks. The discrete spheres formed by the transformed variants were subjected to the single sphere cloning in 24-well plates. One of the clone cultures established from a single sphere was expanded by two passages before experimentation.

### Growth characteristics and cell size measurements

The P6 cell output was subcultured as per the 3K3D and 4K3D schemes in T25 flasks and counted on each day until confluence. Lag period, doubling time and saturation density were estimated by the standard growth curve method. Similar estimations were made for the cells of P5 and the clone after plating them at a density of 3000 cells per cm^2^. The saturation density of 3K3D and 4K3D cells was additionally estimated in T75 flasks.

The 3^rd^ day cells were analyzed for cell size using Cellometer Vision (Nexcelom Bioscience LLC, Lawrence, MA, USA) which was previously calibrated with beads of known sizes. In brief, the trypsinized cells were pipetted into the disposable counting chambers, bright-field images were automatically captured and the pixel area of the captured cells was converted to corresponding cell diameter.

### Growth arrest of 3T3

The cultures intended for growth arrest were treated for two hours with 4 μg per ml MC (Catalogue No. M4287, Sigma-Aldrich) which was prepared in 3T3-CBS medium by diluting a stock solution of 200 μg per ml of HEPES Buffered Earl’s Salt (HBES). Subsequently, cultures were washed 3-times with 3T3-CBS medium with 15-minutes incubation at each step, detached using 0.25% trypsin + 0.03% EDTA solution and counted by trypan blue exclusion. The cells were replated alone at a density of 14000 cells per cm^2^ or co-cultured with epidermal keratinocytes in triplicate T25 flasks and incubated in keratinocyte growth medium (KGM) which was renewed every 3 days. The cultures beyond 8^th^ passage were exposed to MC at every passage, washed thrice and continued incubation in KGM. The responsiveness of serial cultures to MC was tested until the cells showed anchorage-independent growth for two consecutive passages.

### Assessment of feeder cell fate

The MC treated cells were microscopically assessed on a weekly basis, assigned as completely disintegrated or proliferating and the duration elapsed was recorded. Criteria considered for complete disintegration were the absence of healthy cells and presence of a very low density of cells that exhibited vacuolated cytoplasm and/or loss of membrane integrity. The visual assessment was performed by three observers. Complete disintegration was verified by transferring the remaining attached cells by trypsinization to a fresh flask and observing for cell attachment. Conversely, cultures designated as proliferating if one or more proliferative foci were observed were continued incubation until confluence.

### Anchorage-independent growth assay

10^3^ to 10^5^ 3T3 cells at every passage were suspended in methylcellulose (Methocel, Sigma-Aldrich) which was prepared at a final concentration of 0.8% in 3T3-CBS medium, poured over a base of 0.6% agar in 35 mm dish and incubated under standard culture conditions in triplicate for 2 weeks with periodic examination under Nikon inverted phase contrast microscope.

### Keratinocyte—feeder co-culture

Primary Human epidermal keratinocytes (Cat No.PH10205A, www.genlantis.com) were co-cultured with feeder cells by the basic Rheinwald-Green (1975) technique. The keratinocyte growth medium (KGM) consisted of DMEM with 1/3 volume Ham’s F12, 10% FBS, adenine (1.8 x 10^-4^M), transferrin (5 μg/ml), triiodo-L-thyronine (2 x 10^-9^M), dexamethasone (1μg/ml), hydrocortisone (0.4 μg/ml), insulin (5 μg/ml), Cholera Toxin (1 x 10^-10^M), L-serine (20 μg/ml) and L-glutamine (100 μg/ml). EGF (10ηg/ml) was added to the medium 2 days after culture initiation. Flasks containing 14,000 cells per cm^2^ feeder cells were inoculated with 2000 cells per cm^2^ keratinocytes and incubated at standard culture conditions for 7–10 days. The cultures were then treated with 0.03% EDTA to remove feeder cells followed by detachment of keratinocytes using 0.08% trypsin + 0.01% EDTA + 0.025% glucose [1] and the viable cells were counted by trypan blue exclusion.

### Assessment of feeder contamination

The non-proliferative feeder cell contamination was assessed by plating 1000 detached keratinocytes per 60-mm dish and incubated overnight in KGM. To assess proliferative feeder contaminants, 50,000 keratinocytes were plated in T25 flasks and incubated in KGM for 3 weeks. The cultures were fixed in a chilled solution of three parts of methanol and one part of glacial acetic acid stained with 0.125 μg Hoechst 33258 (Sigma, H-6024) per ml of Hank’s balanced salt solution in the dark for 10 minutes, washed with distilled water and observed in an inverted fluorescence microscope (Nikon Diaphot 300) fitted with excitation filter of 330–380 nm and emission filter of 420 nm. The 3T3 cells were distinguished from Keratinocytes on the basis of nuclear size, morphology and fluorescence pattern [12].

### Statistics

The experiments of growth arrest, co-culture, anchorage-independent growth assays and feeder cell contamination assays were performed in triplicate. The cell yields among different groups were compared by Student’s t test while the linearity of cell yields in serial subcultures was tested by regression. Skew, Kurtosis, Confidence level for mean of cell size distribution were calculated in Microsoft Excel (2007).

## Results

### Cell banking

The split ratio that worked out during the establishment of master bank ranged from 1:2 to 1:6.4 while it was 1:8 during expansion for working bank and an average seeding density of 3028±113 cells per cm^2^ was maintained throughout_._

### Subculture schemes and response to MC

**Fig 1:**
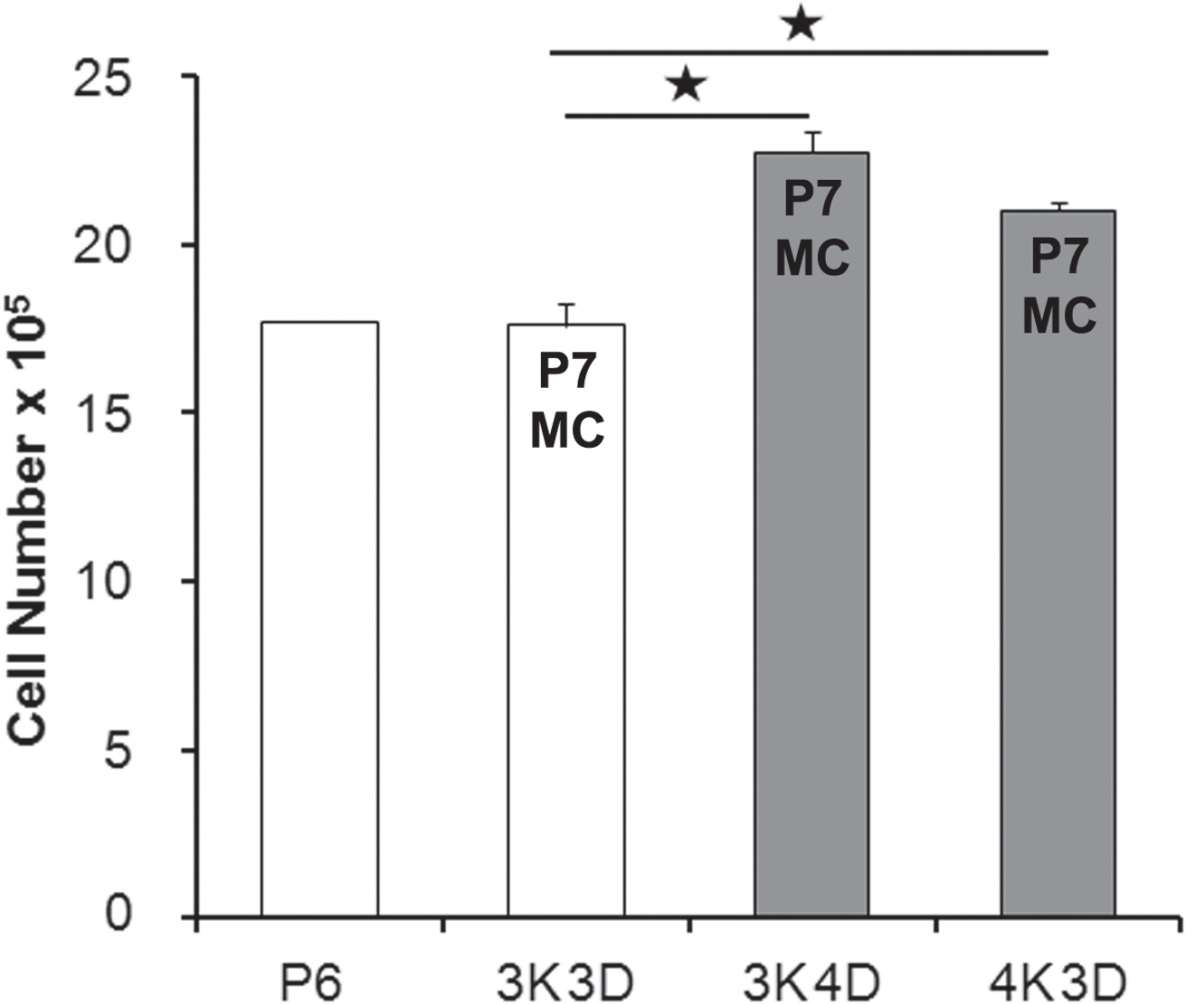
P6 and P7 cell yields and responsiveness to Mitomycin C. Column chart representing the influence of subculture schemes on Swiss 3T3 cell outputs in T75 flasks at 7^th^ passage (P7) and responsiveness to Mitomycin C (MC). The uniformly set up 6^th^ passage (P6) cultures from a working bank were subcultured to P7 by schemes of 3K3D, 3K4D and4K3D (* P<0.05). Shaded columns represent exhibition of resistance to MC and each column represents a mean value from 3 flasks with the standard deviation.

The P6 cultures yielded 1,771,851 ± 36,695 cells that corresponded to 40% confluence. The subsequent subcultures as per 3K3D, 3K4D and 4K3D schemes yielded 1,759,950 ± 64,089, 2,276,700 ± 63,580 and 2,103,675 ± 27,152 cells, respectively (Fig. 1) which corresponded to confluence levels of 42%, 52% and 48%, respectively. As expected for the higher seeding or longer incubation, the P7 cell yields of 4K3D and 3K4D schemes, were significantly higher (P<0.05) than the 3K3D scheme.

**Fig 2:**
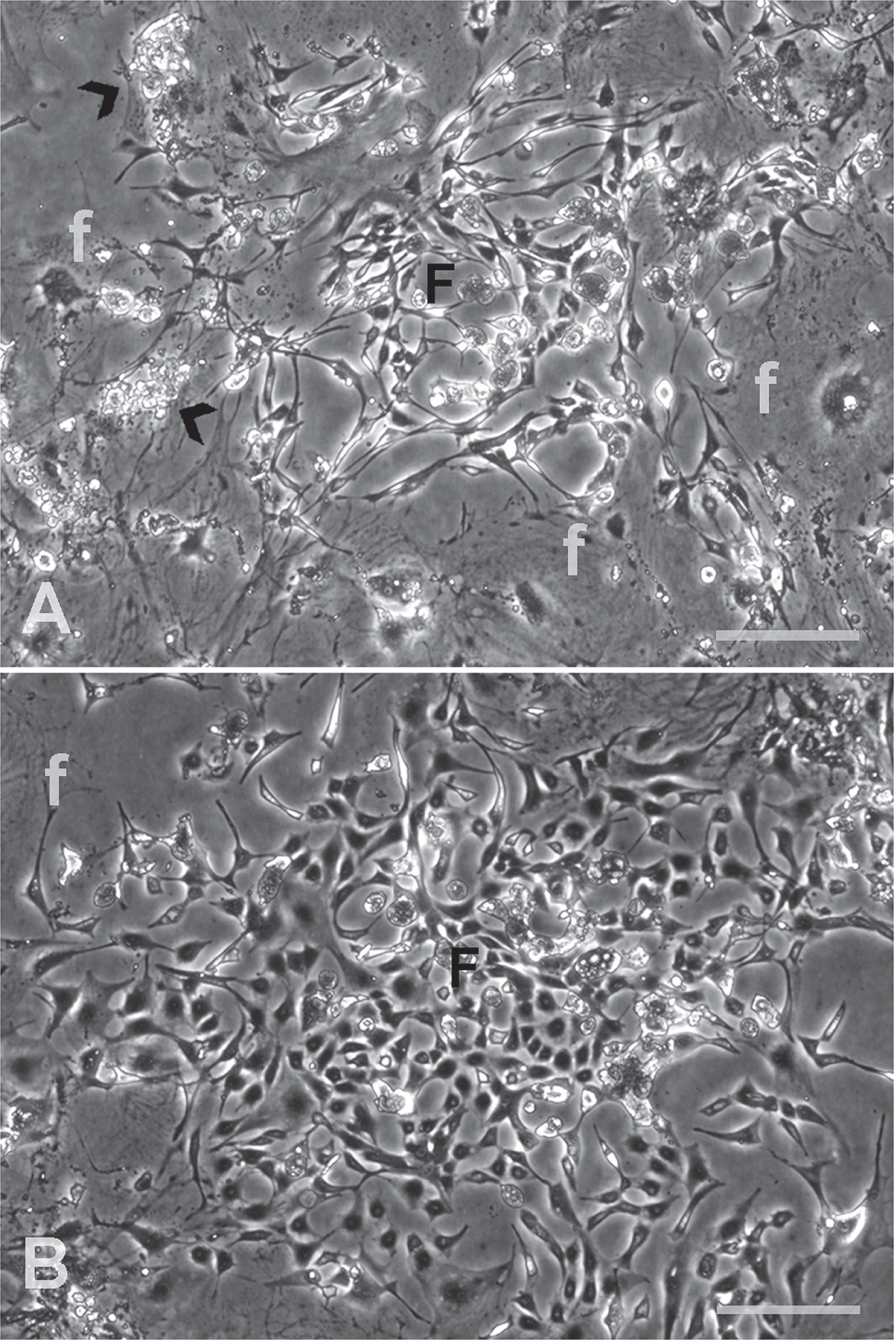
Feeder regrowth in 3K4D culture after mitomycin C treatment. The newly formed bipolar cells (F) appeared amidst the attached MC treated large cells (f) and the floating cellular debris (arrowhead, A) after four weeks of post-treatment period. Five weeks later, the same field showed a distinct large collection of multipolar cells with high nucleus to cytoplasm ratio (B). Scale bar: 100 μm.

There was a resumption of cell division in MC treated 3K4D and 4K3D flasks as evident by the formation of proliferative foci, whereas 3K3D cultures exhibited irreversible growth arrest (Table 1). The formation of proliferation foci in 4K3D cells was rapid which was noticed as early as 1 week after re-plating. However, it was noticed in 3K4D only after 4 weeks by which time the disintegration of susceptible cells was extensive (Fig. 2A). Initially, the foci in reviving cultures were less apparent and showed diffuse zones of bipolar cells among a mix of large attached cells with or without vacuolated cytoplasm and floating cellular debris. Most of such zones, but not all, progressively turned into distinct proliferative foci of polygonal cells with high nucleus to cytoplasm ratio (Fig. 2B).

**Table 1:** Influence of various subculture schemes at P7 of Swiss 3T3 cells on anchorage- independent growth and responsiveness to Mitomycin C (MC) treatment.

**Subculture Scheme**	**Growth in methylcellulose**	**Post-MC incubation period (Weeks)**	**Cells attached after subculture**	**Post-MC status of culture**
**3K3D**	Nil	6	No	D
**3K4D**	Nil	4	-	P
**4K3D**	Nil	1	-	P

D = Disintegrated cultures;

P = Proliferative cultures.

**Fig 3:**
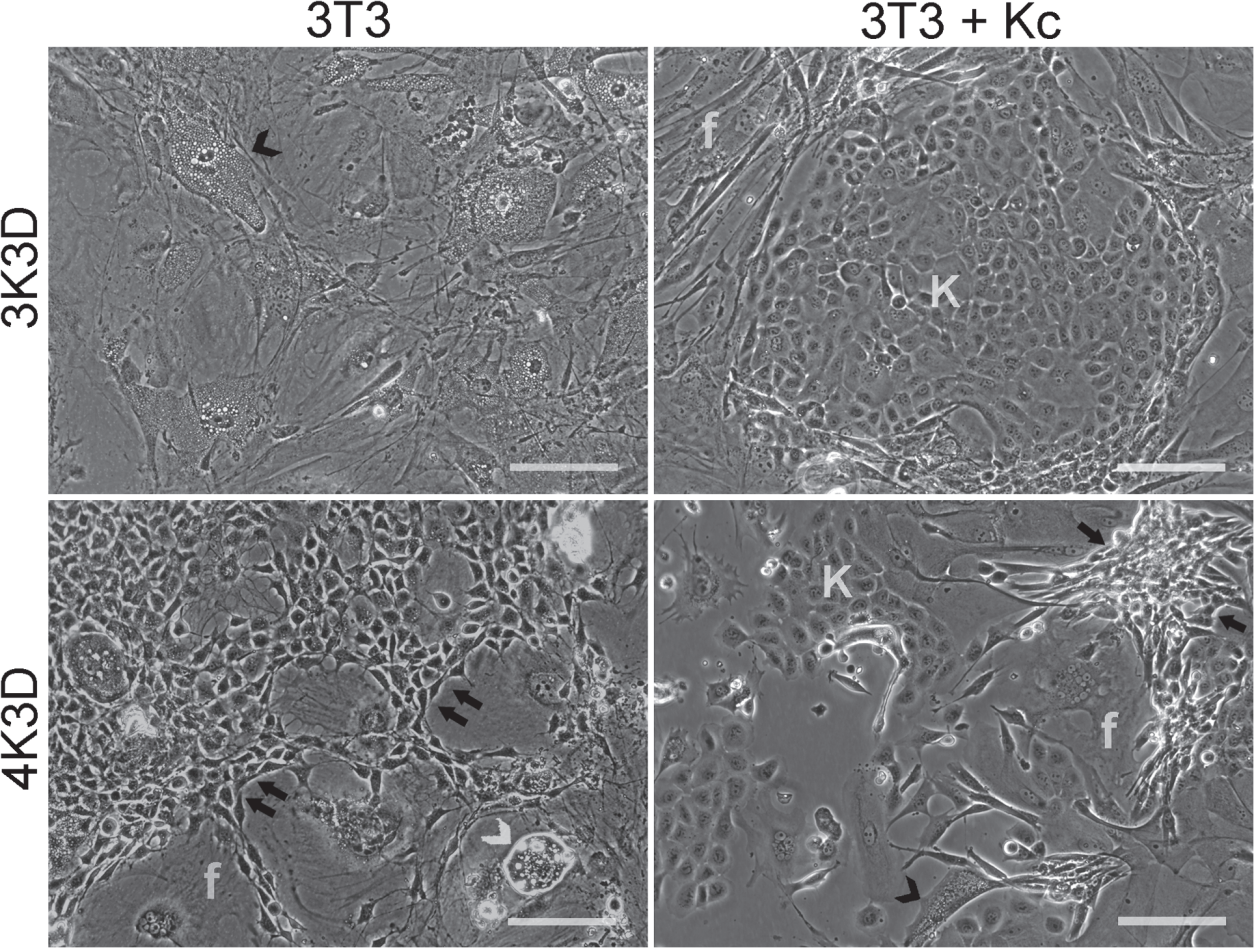
Influence of subculture schemes on responsiveness to Mitomycin C. The 3K3D feeders plated either alone (3T3) or co-cultured with human epidermal keratinocytes (3T3 + Kc) showed degeneration of feeders (arrowhead) and a large well circumscribed keratinocyte (K) colony surrounded by inactivated feeders. The 4K3D feeders plated alone exhibited newly formed compact proliferative feeder cells with high nucleus to cytoplasm ratio (arrows) over a background of enlarged (f) and degenerating cells (white arrow head). The co-culture with human epidermal keratinocytes showed the proliferative foci (arrows). The 3T3 alone and the co-cultures were 2 and 1 weeks old, respectively. Scale bar: 100 μm.

The frequency of proliferative foci at any given time point appeared to vary among replicates. But, it was not possible to accurately estimate the number of foci because they were more frequent at the periphery of culture flasks making the observation difficult. Not all proliferative zones progressed into distinct foci, but a few remained stable or even disintegrated. Several new foci appeared intermittently while a number of old ones grew larger and coalesced. All cultures showing progressive proliferation with the new cellular phenotypes became confluent in additional 8–12 weeks. On the other hand, the MC-exposed fibroblasts from 3K3D showed progressive irreversible disintegration and presented extensive vacuolization and completely disintegrated in 6 weeks (Fig. 3 top left; Table 1). So far, we have validated the 3K3D subculture scheme on 22 out of 40 working bank stocks for irreversible growth-arrest.

### Keratinocyte—feeder co-culture

One week old co-cultures initiated with the 3K3D feeder cells revealed discrete keratinocyte colonies surrounded by the non-proliferating feeder cells which presented signs of disintegration (Fig. 3). But the 4K3D feeders that showed failed growth arrest as early as one week after they were plated alone, similarly revived and produced several proliferative foci in co-cultures. The newly proliferated 3T3 fibroblasts were easily identified by their characteristic narrow cell bodies compared with the large disintegrating cells that presented cytoplasmic vacuolization or nuclear fragmentation (Fig. 3 bottom left).

### Feeder cell contamination

**Fig 4:**
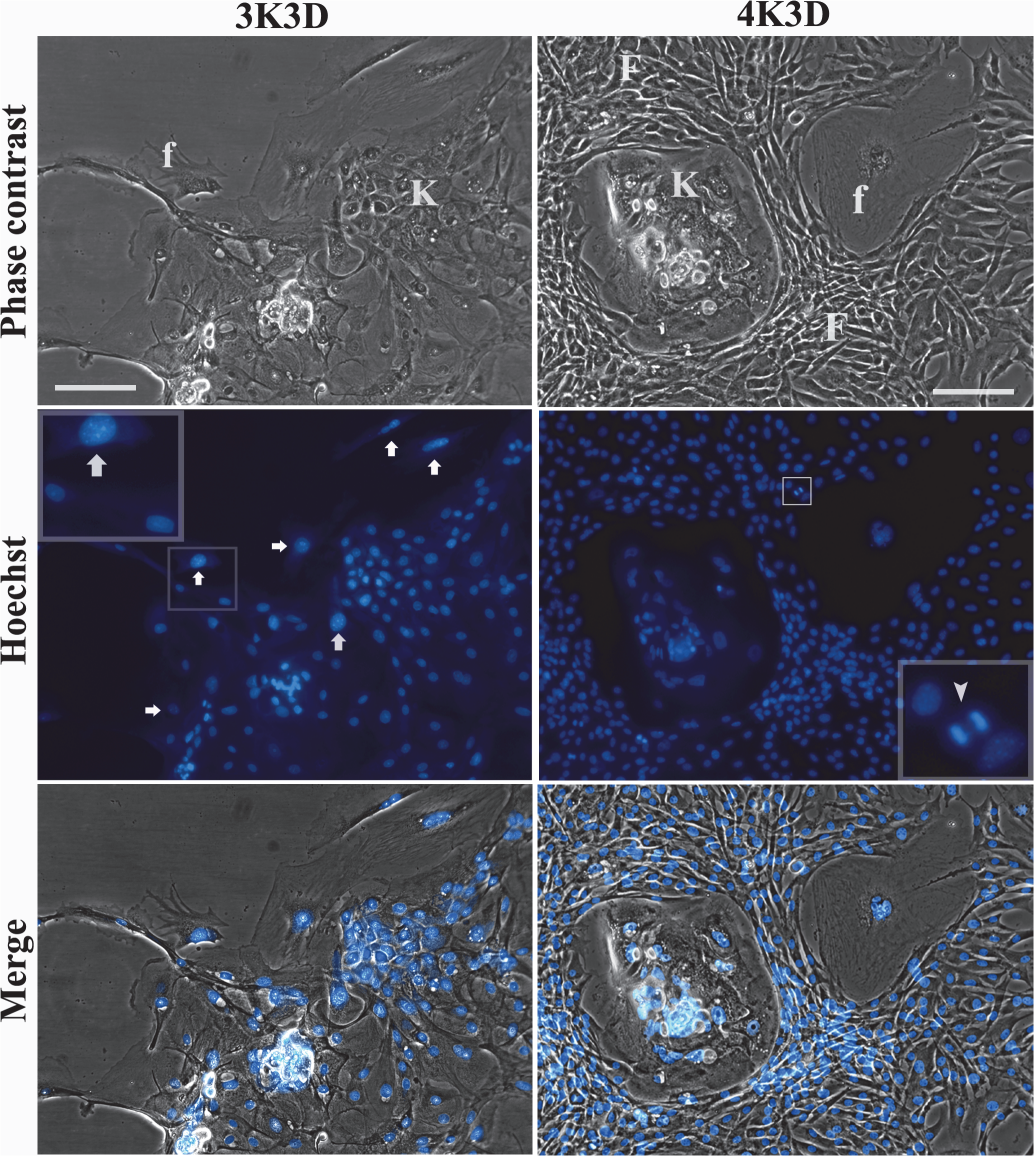
Proliferative feeder cell contamination. Keratinocyte cultures plated alone without the feeders showing cytological features of attached keratinocytes (K) and the contaminating non-proliferative (f) and proliferative feeders (F). The 3K3D feeder cells that came along with the keratinocytes as contaminants (Left panel) presented no dividing cells and were broad with large nuclei in phase contrast and showed coarse chromatin aggregates (arrows) that stained bright in Hoechst (inset of Hoechst image). The keratinocytes comprised of a mix of small polygonal or broad terminally differentiated cells which presented the dull small nuclei. The 4K3D contaminating feeder cells (Right panel) consisted of well circumscribed keratinocyte colonies (K) enveloped by numerous newly formed narrow-bodied feeder cells (F) with several cell divisions (inset of Hoechst image) in addition to a few broad non-proliferating feeder cells (f) showing vesicular nuclei. Scale bar: 100 μm; Left inset: 200 μm; Right inset: 450 μm.

The Hoechst-stained feeder contaminants displayed large nuclei with brightly stained coarse chromatin as compared with the small and dull nuclei of keratinocytes (Fig. 4). The frequency of non-proliferative feeder contamination with 3K3D and 4K3D feeders was uniform at 28 ± 4 per 1000 cells counted. However, when the proliferative capability was assessed, it was found that the 3K3D contaminants progressively disintegrated, whereas the keratinocytes showed a limited proliferation (Fig. 4 Left panel). In contrast, the 4K3D feeder contaminants proliferated to generate a large population of small cells (Fig. 4 Right panel). The invading new feeder cells, presenting several dividing cells, tightly enveloped the keratinocyte colonies and the few large non-proliferative feeder cells.

### Anchorage-independent growth

**Fig 5:**
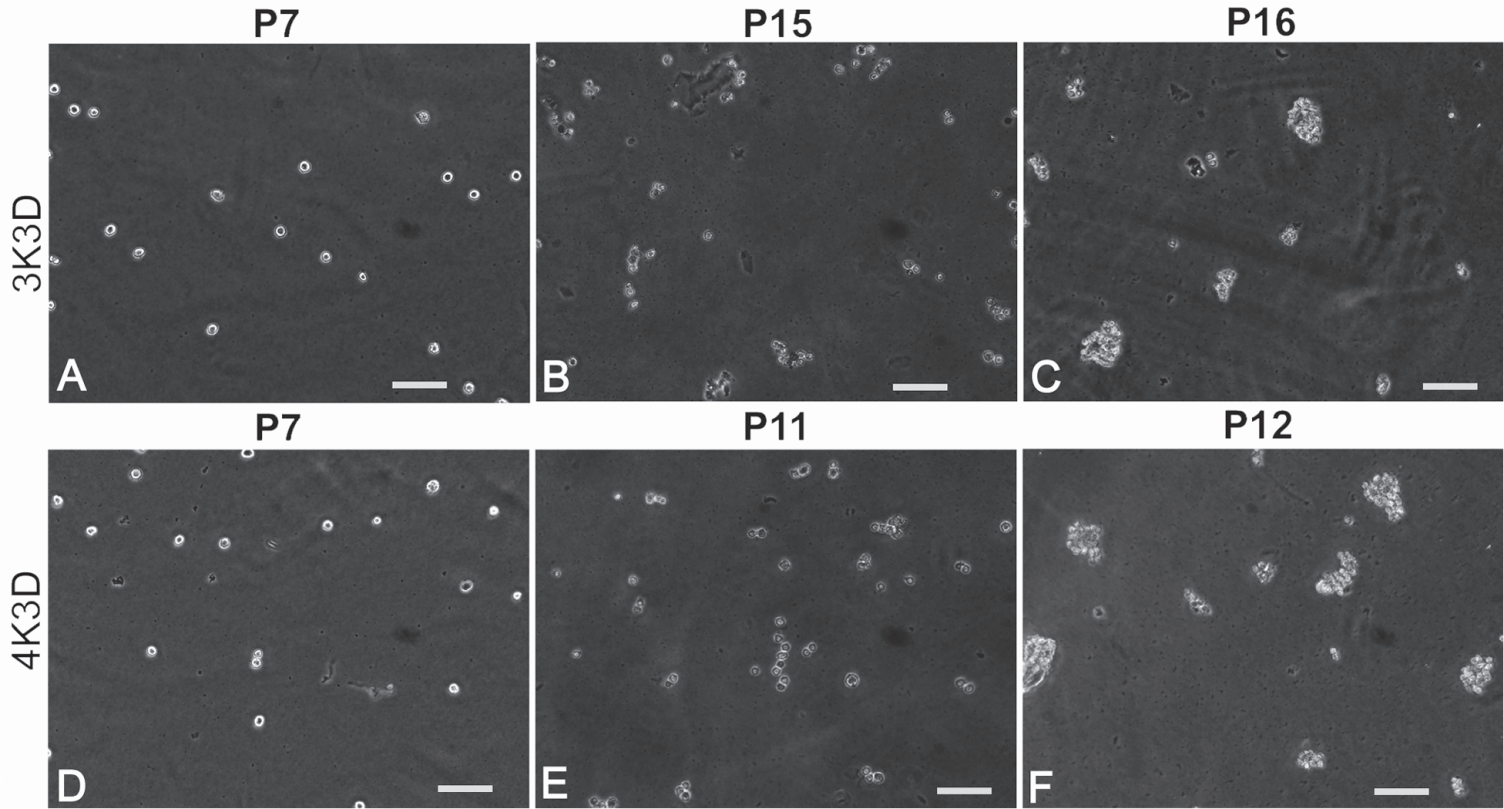
Anchorage-independent growth assay. Swiss 3T3 fibroblasts from different passages during serial subculture by the 3K3D and 4K3D schemes were plated in methylcellulose. Discrete cells from 7^th^ Passage (P7) cultures of 3K3D (A) and 4K3D (D) subculture schemes showed smooth outline. Cells with angular outlines having a shallow contrast were observed at P15 of 3K3D (B) and P11 of 4K3D (E) with occasional linear aggregates. Conspicuous 3-dimensional spheres appeared in the subsequent passages of P16 (C) and P12 (F). Scale bar: 100 μm.

No cultures until P7 showed anchorage-independent growth in any of the dishes of methyl cellulose (Table 1). Similarly, the serially passaged cultures from P8 to P14 of 3K3D scheme and P8 to P10 of 4K3D scheme failed to grow in suspension (Table 2). However, the 3K3D cells showed linear and 3-dimensional growth at P15 and P16, respectively (Fig. 5) while the 4K3D cells produced similar growth at earlier passages of P11 and P12, respectively. The cells of these cultures exhibited angular outlines with a shallow contrast, as opposed to the smooth outlines of single cells in non-proliferating cultures. The growth of cells in dishes plated with 50,000 cells was observed within 4–6 days of incubation as compared with two weeks in at least one of the dishes plated with 1000 cells.

**Table 2:** Influence of serial subculture schemes at various passages of Swiss 3T3 cells on anchorage-independent growth and responsiveness to Mitomycin C (MC) treatment.

**Subculture Scheme**	**Passage**	**Growth in methylcellulose**	**Post-MC incubation period (Weeks)**	**Cell attachment after subculture**	**Post-MC status of culture**
**3K3D**	8	Nil	8	No	D
	9–11	Nil	10	No	D
	12	Nil	11	-	P
	13	Nil	19	Yes*	D
	14	Nil	4	-	P
	15	Linear	3	-	P
	16	3-D	2	-	P
**4K3D**	8–10	Nil	1	-	P
	11	Linear	1	-	P
	12	3-D	1	-	P

D = Disintegrated cultures;

P = Proliferative cultures;

* Few loosely attached vacuolated cells completely disintegrated in 7 days.

### Serial subculture and response to MC

**Fig 6:**
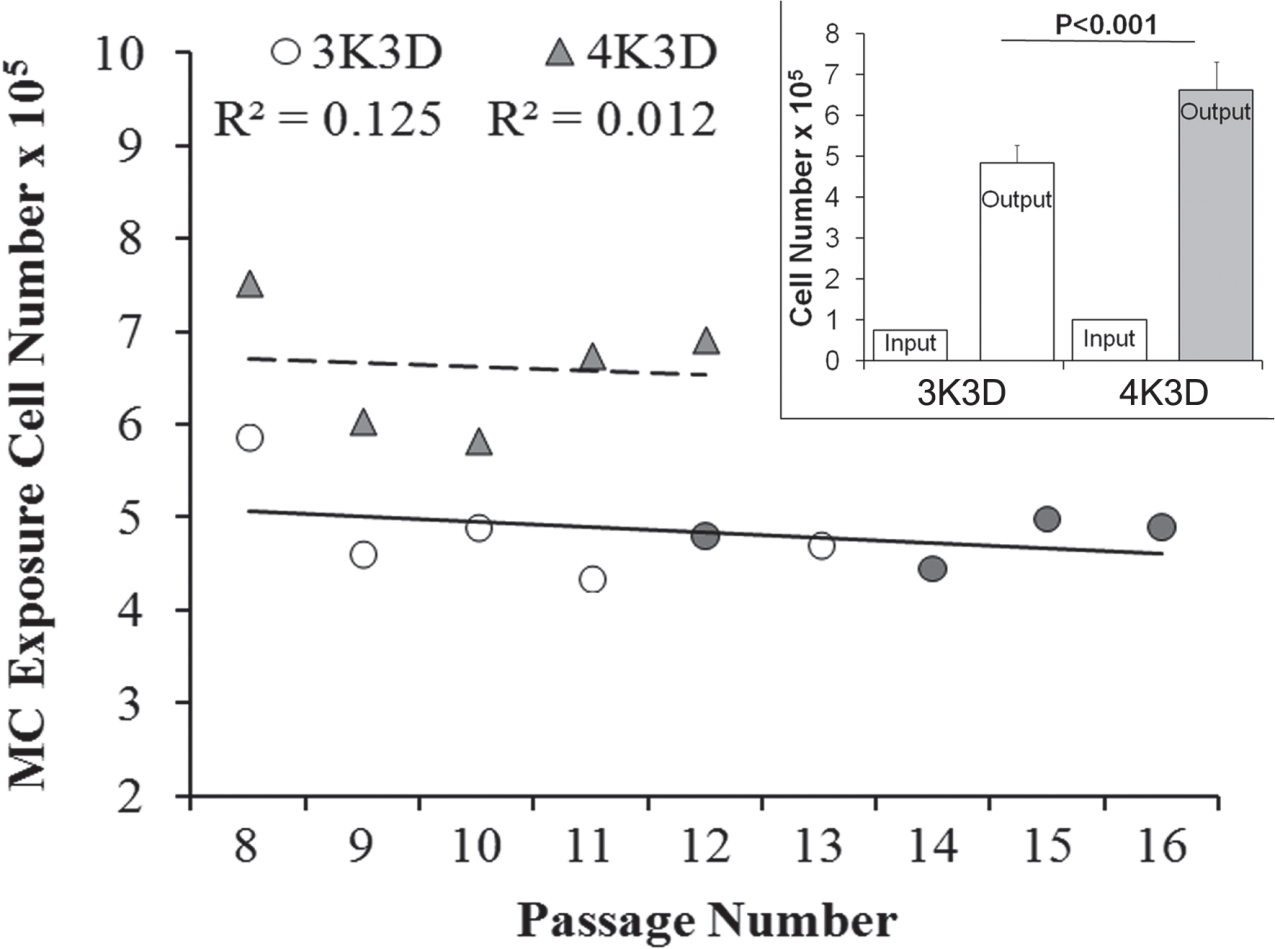
Cell yields during serial subculture. Scattered plot showing the **i**nfluence of serial subculture of Swiss 3T3 fibroblasts by schemes of 3K3D and 4K3D on cell outputs and responsiveness to Mitomycin C treatment at each passage. The 3K3D cultures were serially subcultured either by the same scheme or the 4K3D scheme. Shaded markers denote passages at which MC treated cells showed proliferative foci. The columns in the inset represent the number of cells seeded (Input) and yield (Output). Each column represents average cell number of all the passages from each scheme. The shaded column represents consistent resistance to Mitomycin C at all passages.

The cell yields of serial cultures passaged by either 3K3D (R^2^ = 0.125) or 4K3D (R^2^ = 0.012) scheme did not exhibit any significant trend (Fig. 6). The average 4K3D cell output of 6.62 ± 0.69 x 10^5^ was significantly (P<0.001) higher than 4.84 ± 0.44 x 10^5^ of 3K3D (Fig. 6 inset) and these values corresponded to 72% and 51% confluence, respectively.

The serial cultures of the 3K3D scheme from P8 to P11 exposed to MC exhibited total cellular disintegration that took 8–10 weeks (Table 2). But the subsequent subcultures exhibited the conspicuous proliferation foci with the exception of P13, which disintegrated slowly over more than 19 weeks of post-MC incubation. The time period for the proliferating cells to appear during post-MC incubation gradually decreased with increase in passage number. It was 11, 4, 3 and 2 weeks for P12, P14, P15 and P16 cultures, respectively. On the other hand, the 4K3D cultures persistently resumed post-MC cell proliferation at all the tested passages within a week.

### Transformed clone and response to MC

**Fig 7:**
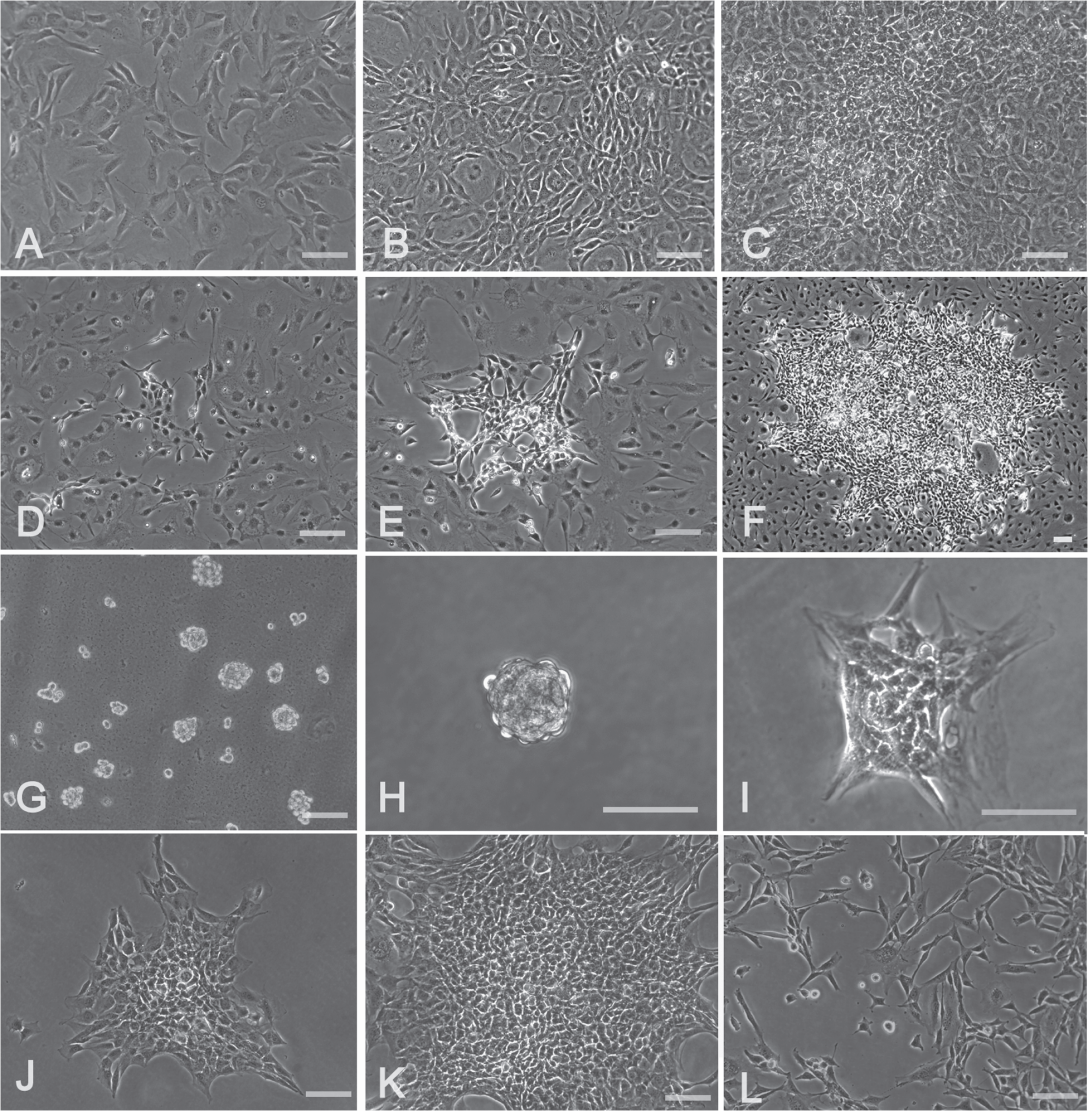
Establishment of spontaneously transformed clone. The culture of 7^th^ passage Swiss 3T3 fibroblasts initiated with 3000 cells per cm^2^after 3 days showed cells with uniform morphology (A) which reached confluence in five days (B) and formed few transformation foci in ten days (C). After two weeks, the cells from such foci were scraped, trypsinized briefly and the single cells were re-plated in a fresh flask. Several discrete clusters of small cells with compact cell bodies and high nucleus to cytoplasm ratio appeared in two days (D) which grew steadily forming conspicuous colonies in four days (E) and enlarged further in ten days (F) over a background of normal looking broad cells. Two weeks later, the trypsinized cells from this culture formed discrete spheres in methyl cellulose (G) which were subjected to the single sphere cloning in 24-well plates (H). The spheres readily anchored overnight (I), spread out to form small colonies in three days (J) and established as large dense colonies in ten days (K). One such colony further expanded in flasks presented narrow bipolar and tripolar cells (L). Scale bar: 100 μm.

A clone was established from a transformation focus in a super-confluent culture. Initially, few foci of heterogeneous cells appeared in a 3K3D culture after two weeks of incubation in FBS containing medium (Fig. 7 A-C). The cells from a large focus isolated by scraping and trypsinization were able to induce more of such foci in a subsequent culture. Each focus was a discrete collection of small cells characterized by high nucleus to cytoplasm ratio (Fig. 7 D-F). The single cell suspension from this culture incubated in methyl cellulose grew into three-dimensional spheres (Fig. 7 G). A single cloned sphere produced a large homogeneous colony with irregular margins after ten days of incubation (Fig. 7 H-K). The colony was further expanded by two subcultures in culture flasks. The resultant cell population predominantly consisted of narrow bipolar and tripolar cells (Fig. 7 L).

**Fig 8:**
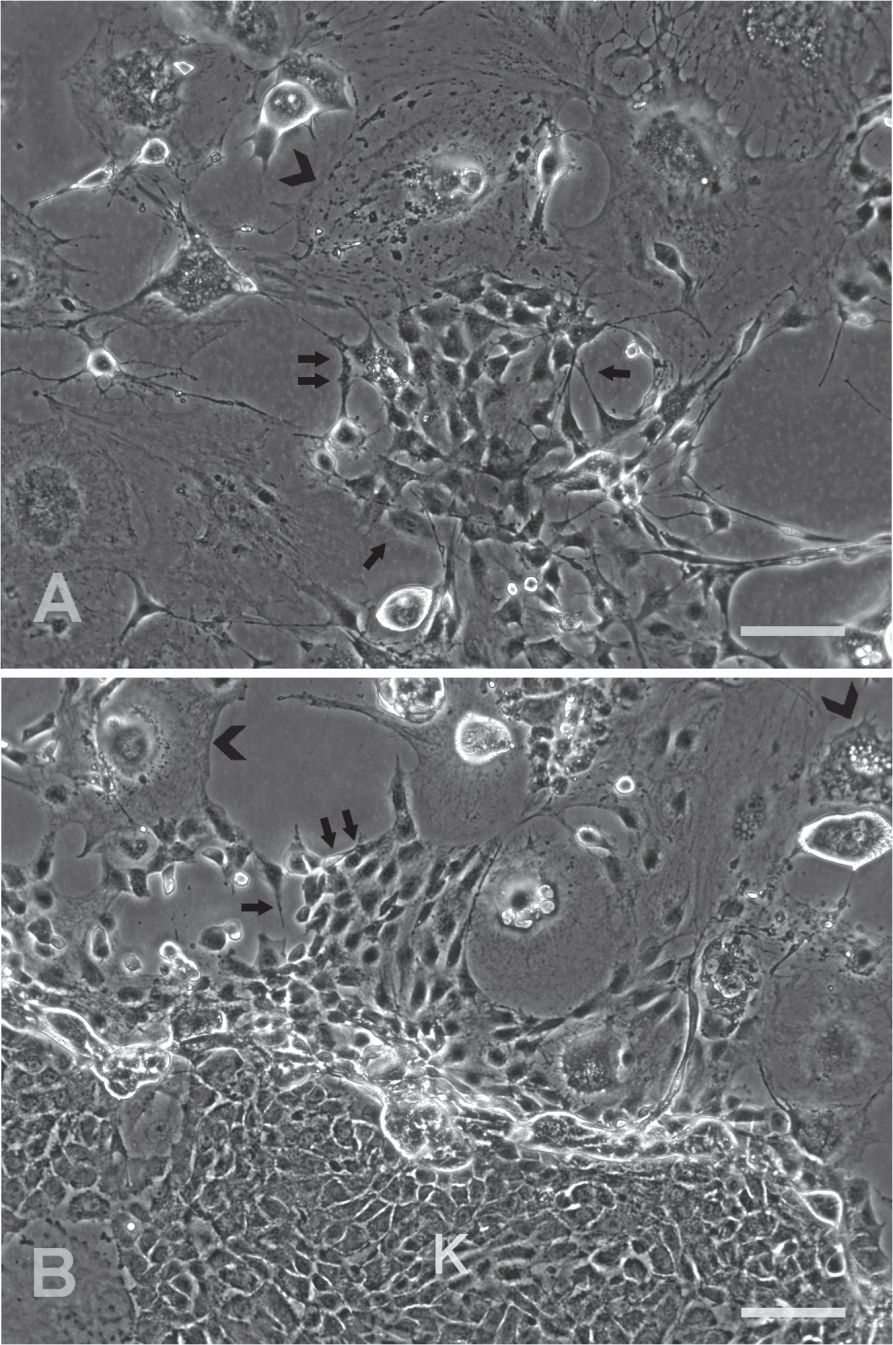
Failure of growth arrest in spontaneously transformed clone. The 2^nd^ passage culture of the clone exposed to Mitomycin C and replated alone (A) exhibited the proliferative focus consisting of the newly formed compact cells (small arrows) surrounded by the broad cells (arrowhead) after 14 days of incubation. The co-culture of the growth-arrested clone cells with the human epidermal keratinocytes (B) presented the well-spread out degenerating cells (arrowhead) and the newly formed cells (small arrows) at the periphery of a large colony of keratinocytes (K) after 7 days. Scale bar: 100 μm.

The MC-exposed clone cells after two weeks showed several pockets of newly formed small cells in the midst of enlarged and degenerating cells (Fig. 8 A). Similarly, the epidermal keratinocytes co-cultured with the MC-treated clone cells showed the presence of the newly proliferating and the disintegrating cells. Both the cell types were easily distinguishable from the discrete keratinocyte colonies within a week under the phase contrast (Fig. 8 B).

### Growth characteristics

**Fig 9:**
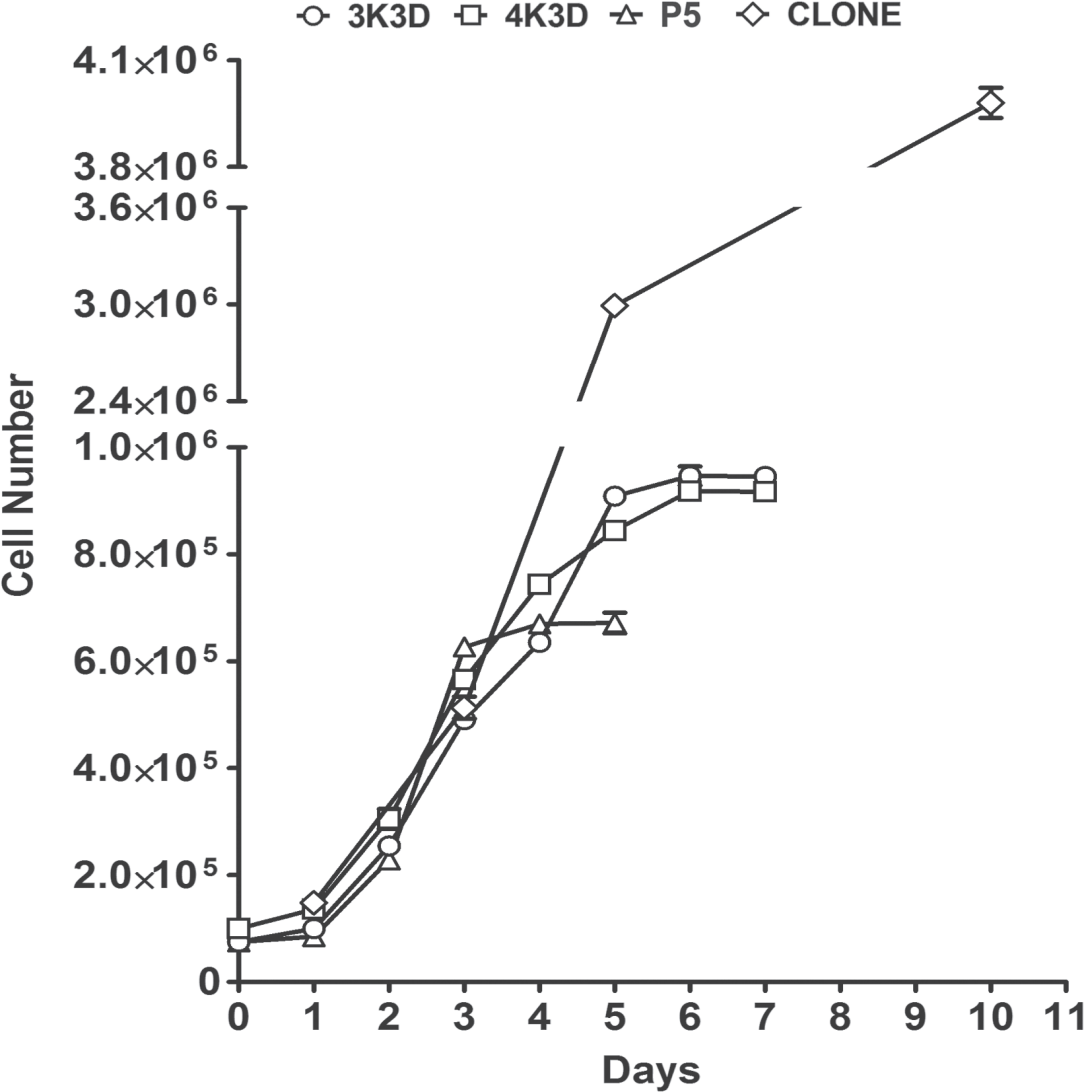
Growth curves. Growth curves of Swiss 3T3 cells of P5, spontaneously transformed clone and the P7 cells produced by subculture schemes of 3K3D and 4K3D.

The growth curves of 3K3D and 4K3D were more or less similar (Fig. 9). The lag phases of 3K3D and 4K3D cells were 32.4 and 30.0 hours, respectively; the doubling times were 38.2 and 38.5 hours, respectively; the saturation densities in T25 flasks were 37,800 and 36,700 per cm^2^, respectively while they were 1.48 times higher at 56,000 and 1.6 times higher at 58,700 per cm^2^ in T75 flasks, respectively (Table 3). This was in contrast to the P5 cells which exhibited 26.5 hours of lag phase; 17.7 hours of doubling time and T25 saturation density of 26,800 cells per cm^2^, while it was 1.81 times higher at 48,500 in T75. On the other hand, the spontaneously transformed clone cells exhibited the longest lag phase of 67.7 hours with a prolonged pre-exponential phase, a doubling time of 26.7 hours and attained a much higher density of 159,200 cells per cm^2^ after 10 days in culture exhibiting transformation foci.

**Table 3:** Influence of subculture on growth characteristics and cell size in Swiss 3T3 cells.

**Characteristics**		**3K3D**	**4K3D**	**P5**	**Clone**
**Lag phase (Hr)**		32.4	30.00	26.5	67.7
**Doubling time**		38.2	38.5	17.7	26.7
**Saturation density—cells/cm** ^**2**^ **(T25)**		37,800	36,700	26,800	>1,59,200*
**Saturation density—cells/cm** ^**2**^ **(T75)**		56,000	58,700	48,500	
**Cell size (m)**	**Sample size**	1,924	3,053	2,600	2,619
	**Minimum**	5.04	5.04	5.45	5.45
	**Maximum**	33.89	45.17	51.83	51.91
	**Mean**	11.92	15.44	20.48	21.21
	**Median**	11.27	15.13	17.59	17.95
	**Mode**	7.28	14.92	15.40	15.40
	**SD**	4.09	4.19	7.27	7.73
	**Coefficient of variation**	0.34	0.27	0.36	0.36
	**Skew**	1.48	1.86	2.47	2.73
	**Kurtosis**	0.72	2.31	5.8	7.67
	**Confidence Level for mean (95%)**	0.009	0.01	0.009	0.009

* Cell number as on day 10.

### Cell size measurements

**Fig 10:**
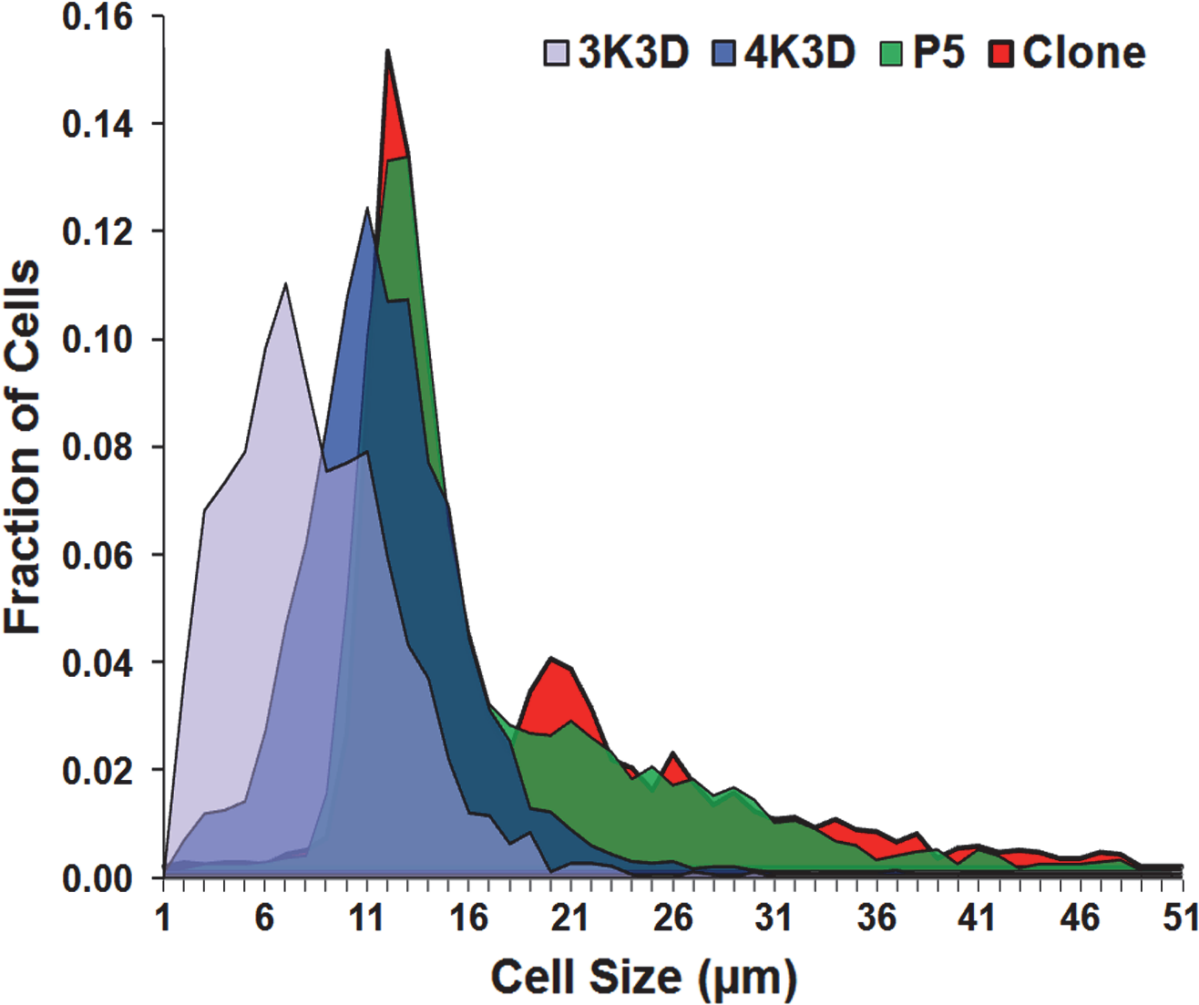
Graphical representation of cell size distribution. Area plot showing Swiss 3T3 cells of P5, spontaneously transformed clone and the P7 cells produced by subculture schemes of 3K3D and 4K3D.

The cell size statistics of 3 days old cultures of 3K3D, 4K3D, clone and P5 cells are given in Table 3. The 3K3D and 4K3D cells showed maximum cell sizes of 33.89 and 45.17, and the mean cells sizes were 11.92 and 15.44, respectively while maintaining a uniform minimum cell size of 5.04 μm. On the other hand, the maximum cell dimensions of P5 and clone cells were higher at 51.83 and 51.91 μm with mean cell sizes of 20.48 μm and 21.21 μm, respectively, while the smallest cell in both the groups measured 5.45 μm. The data from all the groups indicated highly skewed nature of cell size distribution, out of which 3K3D showed the least skewness of 1.48 compared to 1.86, 2.47 and 2.73 for 4K3D, P5 and the clone, respectively. Similarly, the MC sensitive 3K3D cells showed a tendency to a higher uniformity of cell sizes with a smaller kurtosis of 0.72 against 2.31, 5.8 and 7.67 for 4K3D, P5, and the clone, respectively. The deviation in cell size distribution of 3K3D cells compared to other tested groups is apparent from the histogram (Fig. 10).

## Discussion

Murine Swiss embryonic 3T3 cells have been popularly used as feeder cells for large-scale expansion of epidermal keratinocytes for clinical exploitation in the treatment of burns [1, 13, 14]. The basic technique involved co-culture of epithelial cells with the 3T3 feeder cells growth arrested by μ-irradiation or treatment with mitomycin C. The epithelial cells are isolated after selectively removing the persistent feeder cells with EDTA treatment. However, the procedure always allowed a small proportion of feeder cells to remain as contaminants [4, 12]. The problem might be aggravated further if the feeders resumed mitotic activity because foci of proliferation are known to occur in growth arrested murine embryonic fibroblasts [5].

We occasionally observed sporadic formation of such proliferation foci occurring more preferentially in certain feeder batches (Data not shown). We noticed that the different subculture seeding densities employed to generate these feeder batches remained well within the narrow range of supplier’s recommendations. We presumed that the feeder regrowth was perhaps the reflection of subculture dependent distribution of MC resisting cells. The randomness may be due to initial low frequency of such cells which upon serial passaging might become regular. Therefore, we investigated the relationship of the growth arrest failure with the variables of seeding density, the culture duration, and the serial subculture. This study revealed that the MC resistance was specific to subculture procedure. It underlined the need to establish a safe protocol independently for each supplied culture, which possibly contained low level of such variants. The 3T3 cells, originally established by Todaro and Green [15], have been extensively circulated in the world through various repositories for several decades. Each lot of cells has since been repeatedly subcultured leading to the accumulation of variants [8]. It is likely that the cultures resisting growth arrest facilitated a subculture dependent accumulation of specific genetic variants with innate resistance to MC.

Considering that the sensitivity to MC is specific to each feeder cell type [16], the presence of MC-resistant variants may necessitate the use of higher concentrations of MC to ensure irreversible growth arrest. Correspondingly, use of 4 μg/ml was tested to perform optimally in earlier studies [17, 18], while higher concentrations of MC were reported in certain later studies [19, 20, 21, 22, 23]. But the use of higher concentrations can leave harmful residues in target cells [24]. We were able to use a lower concentration yet contained the proliferation of feeder cells by identifying and validating a favorable subculture scheme that perhaps avoided the inclusion of MC-resisting variants.

The other notable point is the induction of clone by prolonged confluence state and exposure to the growth factor rich fetal bovine serum. FBS has been known to stimulate anchorage-independent growth and transformation foci in 3T3 cells [6, 25]. But the use of FBS in Swiss 3T3 cell culture has been widely reported [8, 18, 26, 27] despite the recommendation of the suppliers to use calf bovine serum in culture medium [28]. The existence of several cell lines with the same acronym 3T3 requiring FBS has further complicated the issue.

An important guidance for 3T3 cells is to subculture at 80 percent confluence or less to discourage variant selection [28]. We found that the cultures of all the experimental groups reached a far less confluence state than 80%, yet produced passage scheme dependent feeder regrowth. Another key directive is to limit the passages of 3T3 cells for use in bioengineering of cultured epidermis [29], because the cultured cells respond differently to DNA damaging agents in a passage number dependent manner [30]. Consistently, we found that the serial passaging even by the safer scheme of 3K3D led to MC resistance from P12 onwards, although it was intermittently absent at P13.

There is an utmost need to establish fool-proof growth arrest by observing absolute feeder disintegration because feeder regrowth occurred in P12 cells of 3K3D scheme after 11 weeks, but it took 19 weeks to the P13 cells. Therefore, the standard practice of visually assessing the regrowth of feeders under the microscope for a fixed period of 10–14 days for mitotic activity [5], may not be sufficient to rule out the feeder regrowth. Additionally, it is essential to validate visual observation by transferring the remaining cells for attachment and survival.

Under the normal growth conditions, the 3T3 cells do not show anchorage-independent growth in methylcellulose/soft agar [25]. Therefore, the presentation of three-dimensional growth is indicative of selection of transformants. But this expression was apparent only during late passages as compared with the display of MC resistance at relatively earlier passages. It suggests that the variants responsible for both these presentations are probably not the same. But, on the whole, the methylcellulose assay is of considerable value for different lots of Swiss 3T3 cells as an advance warning for a deviant culture.

The MC sensitive 3K3D cells were the smallest and showed a tendency to decrease in excess asymmetry (skew) in size distribution as compared with the 4K3D cells although both the groups presented comparable growth curves. Moreover, the presentation of the lowest skew and Kurtosis by 3K3D cells suggests phenotypic divergence. Additionally, while considering the doubling time and the saturation density of the P5 cells as near normal [15, 28], the 3K3D cells presented the highly deviated growth characteristics and the cell size distribution parameters. It suggests that the cells of 3K3D, like those of 4K3D and the clone, may not represent a truly normal population. But the presentation of absolute irreversible growth arrest qualifies them as the ideal feeders. It is possible that the 3K3D subculture might have facilitated the accumulation of variants other than those that resisted MC thereby altering the growth characteristics. Normally, the repositories perform batch-testing of cell lines for basic growth characteristics and accordingly issue guidance on subculture procedures [28], but do not test for MC resistance. Therefore, several combinations of the recommended seeding densities and incubation periods need to be included during subculture and tested for MC resistance. Once a validated procedure is established it is likely to work safe for the rest of the cell batches generated by a two-tiered banking system [29].

Lastly, the other candidate feeder cell types which are currently in use may also need to be evaluated similarly for responsiveness to growth arresting methods including μ-irradiation because growth arrest failure is also known to occur in such cells [5].

## Conclusion

We recommend the adoption of tests to rule out (1) MC-resistance (2) proliferative feeder cell contamination of target cells and (3) growth in methylcellulose before qualifying the feeder batches as safe. The subculture of the already over passaged Swiss 3T3 cells should be limited and use of FBS in place of bovine calf serum is an avoidable risk factor. Additionally, the MC resisting clone and the late passage cultures are useful to study molecular pathways involved in chemoresistance and post-chemotherapy initiation of cell cycling in cancer cells.

## Supporting Information

S1 Fig The adopted two-tiered banking system. Flow chart illustrating establishment of a two-tiered banking from Swiss 3T3 cells (CCL-92 of ATCC) supplied as a cryo-vial at 115^th^ passage, designated as zero passage and expanded through additional 6 passages at our laboratory.
(TIF)

S2 Fig Experimental Design. Flow chart depicting the experimental approach of testing the influence of varied subculture schemes in Swiss 3T3 cells at the denoted passage number (P). P6 cultures were uniformly set up by incubating 3000 cryo-preserved working bank (WBP6) cells plated per cm^2^ for 4 days in 3T3-CBS medium. The subsequently initiated P7 cultures were subcultured by three schemes. They were 4K3D and 3K3D, representing 3 days incubation of 4000 and 3000 cells plated per cm^2^, respectively and 3K4D denoting 4 days incubation of 3000 cells plated per cm^2^. Parallel cultures from each scheme were tested for anchorage-independent growth in methyl cellulose (M) and responsiveness to Mitomycin C (MC). Subsequently, the 3K3D cells that exhibited no resistance to MC were further serially subcultured as per 3K3D and 4K3D schemes until the presentation of growth in methylcellulose while simultaneously testing for resistance to MC in parallel cultures. A separate P7 culture was grown in 3T3-FCS medium for 2 weeks and subcultured once to induce transformation foci which formed spheres in methylcellulose. A culture established by single sphere cloning was tested for MC resistance. The MC treated cells of 4K3D, 3K3D, and the clone were used to co-culture with epidermal keratinocytes (Kc+F).
(TIF)
