# Supplementary figures and images for: Occurrence and Control of Sporadic Proliferation in Growth Arrested Swiss 3T3 Feeder Cells
Source: PLoS One. 2015 Mar 23;10(3):e0122056. doi: 10.1371/journal.pone.0122056 (PMC4370869; doi:10.1371/journal.pone.0122056)

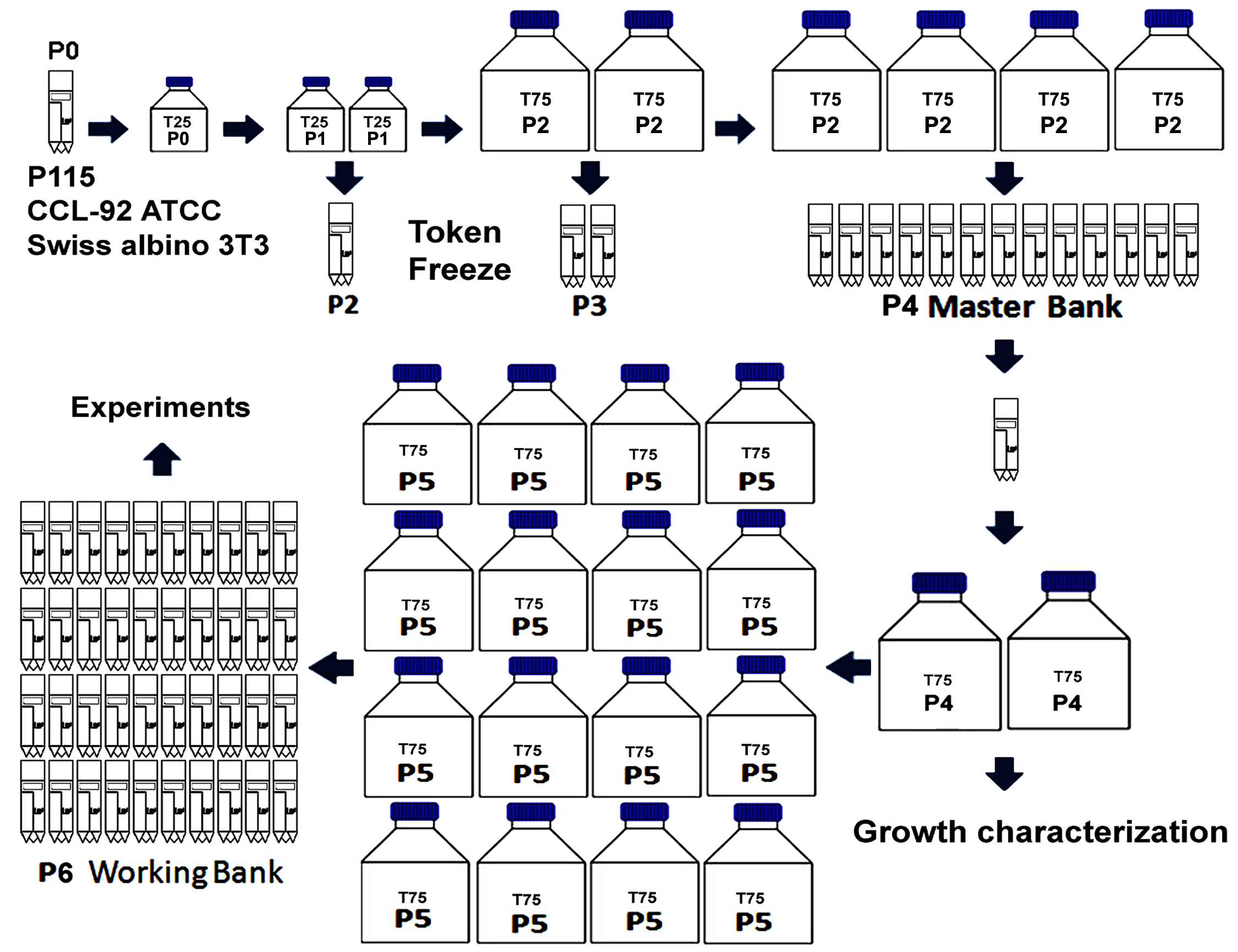

Supplement: S1 Fig — Flow chart illustrating establishment of a two-tiered banking from Swiss 3T3 cells (CCL-92 of ATCC) supplied as a cryo-vial at 115th passage, designated as zero passage and expanded through additional 6 passages at our laboratory. (TIF) [file pone.0122056.s001.tif]

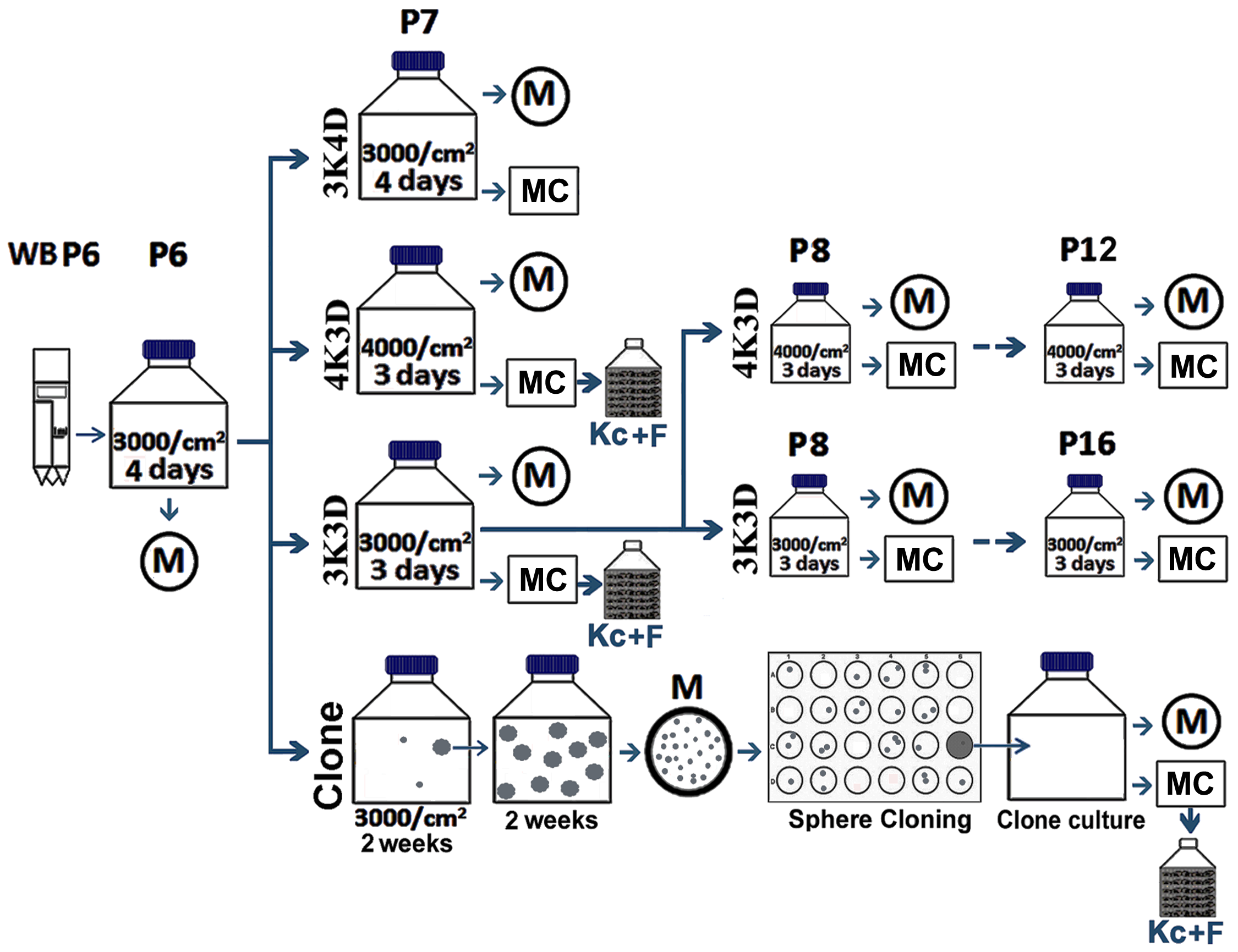

Supplement: S2 Fig — Flow chart depicting the experimental approach of testing the influence of varied subculture schemes in Swiss 3T3 cells at the denoted passage number (P). P6 cultures were uniformly set up by incubating 3000 cryo-preserved working bank (WBP6) cells plated per cm2 for 4 days in 3T3-CBS medium. The subsequently initiated P7 cultures were subcultured by three schemes. They were 4K3D and 3K3D, representing 3 days incubation of 4000 and 3000 cells plated per cm2, respectively and 3K4D denoting 4 days incubation of 3000 cells plated per cm2. Parallel cultures from each scheme were tested for anchorage-independent growth in methyl cellulose (M) and responsiveness to Mitomycin C (MC). Subsequently, the 3K3D cells that exhibited no resistance to MC were further serially subcultured as per 3K3D and 4K3D schemes until the presentation of growth in methylcellulose while simultaneously testing for resistance to MC in parallel cultures. A separate P7 culture was grown in 3T3-FCS medium for 2 weeks and subcultured once to induce transformation foci which formed spheres in methylcellulose. A culture established by single sphere cloning was tested for MC resistance. The MC treated cells of 4K3D, 3K3D, and the clone were used to co-culture with epidermal keratinocytes (Kc+F). (TIF) [file pone.0122056.s002.tif]
